# Analysis of GATA transcription factors and their expression patterns under abiotic stress in grapevine (*Vitis vinifera* L.)

**DOI:** 10.1186/s12870-023-04604-1

**Published:** 2023-12-02

**Authors:** Xiuming Zhang, Jiahui Ma, Shijin Yang, Wenkong Yao, Ningbo Zhang, Xinyi Hao, Weirong Xu

**Affiliations:** College of Enology and Horticulture, Ningxia University/College of Modern Grape and Wine Industry/Ningxia Grape and Wine Research Institute/Engineering Research Center of Grape and Wine, Ministry of Education, Yinchuan, 750021 P. R. China

**Keywords:** Grapevine, GATA family, Transcription factor, Abiotic stress, Expression patterns

## Abstract

**Background:**

GATA transcription factors are type IV zinc-finger proteins that play key roles in plant growth and responses to environmental stimuli. Although these proteins have been studied in model plants, the related studies of GATA gene family under abiotic stresses are rarely reported in grapevine (*Vitis vinifera* L.).

**Results:**

In the current study, a total of 23 *VviGATA* genes were identified in grapevine and classified into four groups (I, II, III, and IV), based on phylogenetic analysis. The proteins in the same group exhibited similar exon–intron structures and conserved motifs and were found to be unevenly distributed among the thirteen grapevine chromosomes. Accordingly, it is likely that segmental and tandem duplication events contributed to the expansion of the *VviGATA* gene family. Analysis of *cis-*acting regulatory elements in their promoters suggested that *VviGATA* genes respond to light and are influenced by multiple hormones and stresses. Organ/tissue expression profiles showed tissue specificity for most of the *VviGATA* genes, and five were preferentially upregulated in different fruit developmental stages, while others were strongly induced by drought, salt and cold stress treatments. Heterologously expressed VamGATA5a, VamGATA8b, VamGATA24a, VamGATA24c and VamGATA24d from cold-resistant *V. amurensis* ‘Shuangyou’ showed nuclear localization and transcriptional activity was shown for VamGATA5a, VamGATA8b and VamGATA24d.

**Conclusions:**

The results of this study provide useful information for GATA gene function analysis and aid in the understanding of stress responses in grapevine for future molecular breeding initiatives.

**Supplementary Information:**

The online version contains supplementary material available at 10.1186/s12870-023-04604-1.

## Background

Plant development and stress responses are regulated by many families of transcription factors (TFs), which control gene expression by binding to specific *cis-*acting regulatory elements in the promoter regions of downstream target genes [1]. GATA factors are evolutionarily conserved TFs that are found in organisms ranging from cellular slime mold to vertebrates, including plants, fungi, nematodes, insects, and echinoderms [2]. Members of the GATA families from animals and yeasts are comparatively small. Only six, eight and four GATA TFs can be identified in human, *Drosophila melanogaster* and *Schizosaccharomyces pombe*, respectively [3]. Most of the animal GATA factors present two zinc fingers, where only the C-terminal zinc finger is involved in DNA binding. The N-terminal zinc finger modulates DNA-binding specificity or mediates the interaction with other proteins [4]. The majority of the fungal GATAs, in contrast, contain a single zinc finger domain and mostly fall into two different categories [5]. In plants, GATA factors contain one conserved type IV zinc-finger motif (C-X_2_-C-X_17-20_-C-X_2_-C) followed by a highly basic region, and bind to the consensus DNA sequence (A/T)GATA(A/G) (WGATAR) in the promoters of their target genes [2, 3]. Structurally, the GATA domain consists of two antiparallel β-sheets, followed by an α-helix and a nonstructured basic tail [4]. Since the first identification of a plant GATA factor, *Ntl1* (NIT2-like) from *Nicotiana tabacum*, GATA TFs have been identified in many plant species, including *Arabidopsis thaliana* (30 members), *Oryza sativa* (28 members), *Solanum lycopersicum* (30 members)*, Malus domestica* (35 members), *Arachis hypogaea* (45 members), *Solanum tuberosum* (49 members) and *Triticum aestivum* (79 members) [3, 4, 6–11]. Based on phylogenetic analysis, and analysis of domain organization and intron–exon structures, the GATA family can be divided into four subfamilies (I-IV), following the organization reported for *A. thaliana* [3].

The biological functions of plant GATA factors have been extensively reported, and include modulation of growth and development, as well as responses to biotic and abiotic stress. For example, *AtGATA2* mediates photomorphogenesis [12], and *AtGATA21/AtGNC* (GATA, NITRATE-INDUCIBLE, CARBON-METABOLISM INVOLVED) and *AtGATA22/AtGNL/AtCGA1* (GNC-LIKE/CYTOKININ-RESPONSIVE GATA FACTOR1) were shown to act downstream from AtARF2 in the control of greening, flowering time and senescence [13]. Other examples include *PdGATA19/PdGNC* from poplar (*Populus deltoides*), which plays a role in photosynthesis and growth [14] and TaGATA1 from wheat (*T. aestivum)*, which modulates seed dormancy and host immune response to the pathogen *Rhizoctonia cerealis* [15, 16]. In rice (*O. sativa*), *OsGATA6* and *OsGATA7* were shown to regulate rice heading, panicle development and grain number per panicle, while OsGATA16 confers cold tolerance by repressing OsWRKY45-1 at the seedling stage [17–19]. Another example of abiotic stress involvement was shown in sweet potato (*Ipomoea batatas*), where IbGATA24 was found to interact with IbCOP9-5a, thereby enhancing drought and salt tolerance [20]. In *Vitis*, it was reported that *GATA2* (named *GATA5a* in this current study) functions as a transcriptional activator and enhances powdery mildew resistance though the involvement of a reactive oxygen species pathway [21]. Additionally, it has also been proposed that plant GATA TFs may have retained ancestral biological functions in the biosynthesis of metal binding complexes, as well as in nitrogen and carbon metabolism [4].

Grapevine (*V. vinifera* L.) is the most valuable horticultural crop in the world [22], the domestication of which occurred concurrently about 11,000 years ago in Western Asia and the Caucasus, to yield table and wine grapes [23]. Nevertheless, with the expansion of areas used for grapevine cultivation, various abiotic stresses including cold, drought and salt are increasingly challenging the grape industry. China is one of the origin of grapevine genus, and has abundant germplasm resources that can be used for *Vitis* breeding [24]. For example, *V. amurensis* is native to north-eastern China and is highly resistant to low temperature, even at -40°C [25]. *V. amurensis* ‘Shuangyou’, which was produced by pistillate flower genotypes as female parents and *V. amurensis* ‘Shuang Qing’ as a male parent for intraspecific crossing, was very interesting due to the hermaphroditic flower and strong cold tolerance [26].

Given their roles in key stress tolerance and associated responses, as well as in fundamental growth processes, there is broad interest in elucidating the functions and potential applications of GATA TFs in horticulturally important crops. In recent years, several reports have demonstrated that a subset of *Vitis GATA* genes are transcriptionally regulated in response to light, phytohormones and biotic stresses [21, 27, 28]. However, the function of GATA factors defined remains very little under abiotic stresses in grapevine. In the current study, we performed a more comprehensive bioinformatics analysis and analyzed the expression profiles of the grapevine GATA gene family under cold, drought and salt stresses, providing valuable information and candidate genes for future molecular breeding in grapevine.

## Results

### Identification of *VviGATA* genes in grapevine

In total, 23 *GATA* genes were identified in the grapevine genome using a Hidden Markov Model (HMM) profile of the GATA domain (PF00320), after Vitvi06g00802.t01 was excluded due to E-values > 1e^−5^, and these were named (Table 1) according to the recently proposed grapevine nomenclature system [29]. Additional information related to the corresponding predicted proteins, including coding sequence (CDS), protein length, molecular weight, isoelectric point, aliphatic index, grand average of hydropathicity (GRAVY) and predicted subcellular localization, is shown in Table 1 and Additional file 1: Table S1. The length of the VviGATA proteins was found to vary from 125 (VviGATA16b) to 735 (VviGATA26) amino acids, which also corresponded to the lowest (14.0 kDa) and highest (84.6 kDa) molecular weight. The isoelectric points of the predicted GATA proteins range between 4.78 and 10.20, with an average of 7.16, showing nearly neutral properties. Notably, the instability index of most VviGATA proteins (21/23) is > 40.00, suggesting that they are unstable. The average aliphatic index was found to be 61.88, ranging from 40.00 to 101.69, reflecting proteins rich in aliphatic amino acids, and the GRAVY < 0.000, with the exception of VviGATA26 (0.065), indicating that they are hydrophilic. Finally, the subcellular localization prediction indicated that 20 VviGATA proteins are localized in the nucleus, and one each in the chloroplast, apoplast and plastid (Table 1).

**Table 1:** Detailed information regarding *VviGATA* transcription factors in grapevine

Gene name	VCost. v3 ID	Chromosome	Protein length	Molecular Weight	Isoelectric points	Instability index	Aliphatic index	Grand average of hydropathicity	Subcellular localization
VviGATA1	Vitvi05g00938.t01	Chr5: 11488096–11490172 (+)	251	28,207.36	8.93	71.75	55.50	-0.884	Nucleu
VviGATA2	Vitvi08g01831.t01	Chr8: 21037320–21039073 (+)	299	33,490.24	5.28	65.26	62.88	-0.877	Nucleu
VviGATA4	Vitvi15g00636.t01	Chr15: 13505444–13506841 (+)	270	29,892.01	6.71	57.84	57.07	-0.753	Nucleu
VviGATA5a	Vitvi03g00037.t01	Chr3: 452645–454156 (+)	317	34,581.26	5.49	71.43	57.54	-0.666	Nucleu
VviGATA5b	Vitvi04g01410.t01	Chr4: 19834595–19836218 (+)	338	36,840.01	5.67	64.11	55.41	-0.696	Nucleu
VviGATA7	Vitvi14g02998.t01	Chr14: 26715212–26732353 (-)	367	40,974.27	7.85	48.86	71.74	-0.711	Nucleu
VviGATA8a	Vitvi06g00271.t01	Chr6: 3427832–3433983 (+)	464	50,520.96	8.14	52.16	62.26	-0.574	Nucleu
VviGATA8b	Vitvi13g00614.t01	Chr13: 5861656–5865169 (-)	340	36,536.18	6.46	66.94	60.82	-0.539	Nucleu
VviGATA9a	Vitvi04g00289.t01	Chr4: 2729898–2731726 (+)	342	37,926.31	5.85	50.05	61.90	-0.650	Nucleu
VviGATA9b	Vitvi09g00311.t01	Chr9: 3439027–3440508 (+)	329	36,379.32	5.87	49.43	55.14	-0.708	Nucleu
VviGATA13	Vitvi06g01610.t01	Chr6: 1526246–1528992 (+)	171	19,120.16	7.64	66.63	40.00	-1.101	Nucleu
VviGATA15	Vitvi07g02214.t01	Chr7: 4011736–4013050 (+)	140	15,404.86	10.20	67.71	73.93	-0.642	Nucleu
VviGATA16a	Vitvi05g00077.t01	Chr5: 747538–748871 (+)	153	16,668.76	9.76	64.31	61.90	-0.918	Nucleu
VviGATA16b	Vitvi14g00123.t01	Chr14: 1203167–1204479 (+)	125	13,989.38	9.76	64.59	63.20	-0.682	Nucleu
VviGATA18	Vitvi04g01299.t01	Chr4: 18788409–18789713 (+)	240	26,788.04	6.40	61.46	56.58	-0.464	Extracellular
VviGATA21	Vitvi11g00180.t01	Chr11: 1817856–1819363 (+)	310	34,297.71	9.31	58.49	57.68	-0.749	Nucleu
VviGATA22	Vitvi04g00111.t01	Chr4: 1062734–1064236 (+)	306	34,091.42	8.76	64.04	49.15	-0.823	Nucleu
VviGATA24a	Vitvi03g01002.t01	Chr3: 14536380–14549507 (+)	302	32,126.58	5.47	44.48	65.86	-0.547	Chloroplast
VviGATA24b	Vitvi03g01766.t01	Chr3: 14596771–14644542 (+)	387	42,646.46	4.86	40.83	63.44	-0.685	Nucleu
VviGATA24c	Vitvi09g01352.t01	Chr9: 20973834–20989595 (-)	299	32,677.24	6.19	37.61	57.09	-0.831	Nucleu
VviGATA24d	Vitvi18g00538.t01	Chr18: 6060270–6094659 (+)	368	40,224.67	4.78	45.79	71.55	-0.594	Nucleu
VviGATA25	Vitvi18g00537.t01	Chr18: 6040085–6058225 (+)	294	32,429.73	5.82	47.89	61.02	-0.761	Nucleu
VviGATA26	Vitvi12g01002.t01	Chr12: 13444491–13447481 (+)	735	84,601.97	9.46	38.93	101.69	0.065	Plastids

### VviGATA phylogeny and conserved domains

**Fig. 1:**
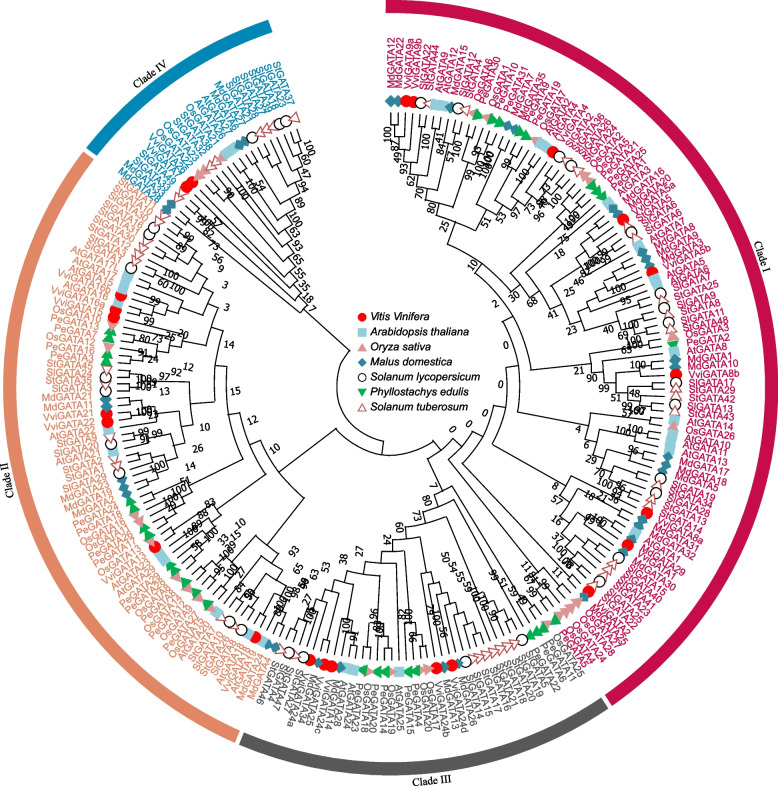
Phylogenetic analysis of GATA proteins from *Vitis vinifera*, *Arabidopsis thaliana*, *Oryza sativa*, *Malus domestica*, *Solanum lycopersicum*, *Phyllostachys edulis* and *Solanum tuberosum*. The phylogenetic tree was constructed based on the full length amino acid sequences (Additional file 2: Table S2) using MEGA 11 with the Neighbor-Joining method and 1,000 bootstrap replicates

**Fig. 2:**
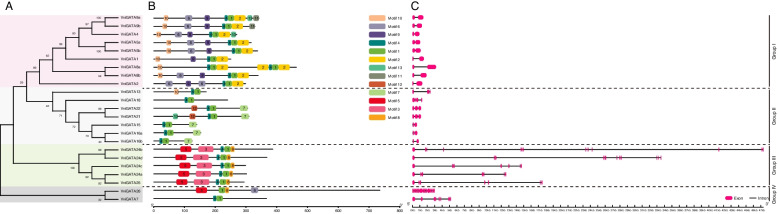
Characterization of GATA genes in grapevine. **A** Phylogenetic relationship between the identified GATA proteins in grapevine. **B** Conserved motif analysis of the VviGATA proteins. The 13 predicted motifs are represented by different colored boxes and the detailed sequence information for each motif is shown in Additional file 5: Table S3. **C** The exon–intron configurations of the corresponding *VviGATA* genes. The closed red boxes and black lines represent exons and introns, respectively

To determine the evolutionary relationships and potential functional divergence of the identified VviGATA proteins, a neighbor-joining phylogenetic tree was constructed based on full-length GATA sequences, including 30 from *A. thaliana*, 28 from *O. sativa*, 30 from *S. lycopersicum*, 31 from *Phyllostachys edulis*, 35 from *M. domestica* and 49 from *S. tuberosum* (Additional file 2: Table S2). This resolved the grapevine GATA proteins into four clades (I-IV; Fig. 1), which corresponded to their assigned phylogeny alone grapevine *VviGATA* genes (Group I-IV) (Fig. 2A). Clade I contained the most members with 9 VviGATA proteins, followed by clade III (7), clade II (5), and clade IV (VviGATA13 and VviGATA18) (Fig. 1). Several grapevine proteins clustered closely with those from *M. domestica* and *A. thaliana*, providing a basis to test for evolutionarily conserved gene function.

All of the grapevine GATA proteins contained only one conserved GATA domain (Additional file 3: Fig. S1), while members in group III also possessed one CCT domain and a TIFY domain, and RPT2 and Bromodomain and extra-terminal (BET) domains were only present in group I (VviGATA8a) and group IV (VviGATA7), respectively (Additional file 4: Fig. S2). Group I, II and IV proteins contained 18 residues between the second and third Cys residues in the zinc finger loop (C-X_2_-C-X_18_-C-X_2_-C), except for VviGATA26, where S-X_2_-C-X_19_-C-X_2_-C replaced C-X_2_-C-X_18_-C-X_2_-C. All 5 group III members contained 20 residues in the zinc finger (C-X_2_-C-X_20_-C-X_2_-C). In addition, several GATA domain amino acids were highly conserved such as GP and LCNACG, although the latter was changed to LCDACG in VviGATA7 (Additional file 3: Fig. S1).

### VviGATA conserved motifs and gene structure analysis

Conserved motifs and gene structures can be used to deduce evolutionary relationships and diversification. 13 motifs were authenticated with E-value < 0.05, including two GATA domains (Motifs 4/1) (Fig. 2B). Motifs 2, 9, and 11 were only observed in group I. Notably, VviGATA8a and VviGATA2 possessed 3 motifs 2 and 2 motifs 6. Motifs 7 and 12 were only identified in Group II members, while motifs 3, 5 and 8 were seen in all Group III proteins, with motifs 5 and 8 also present in Group IV VviGATA26, suggesting that VviGATA26 may have evolved from a Group III gene (Fig. 2B). Motif sequences and logos are listed in Additional file 5: Table S3. Exon–intron analysis revealed that *VviGATA24b* was the longest gene (47.37 Kb), and that Group III and IV genes contained more exons than Group I and II, which had only 2 ~ 4. All Group I members had two exons, while Group II members contained three exons, except for *VviGATA18* that had four exons (Fig. 2C).

### Chromosomal distribution, synteny and tandem duplication analysis

**Fig. 3:**
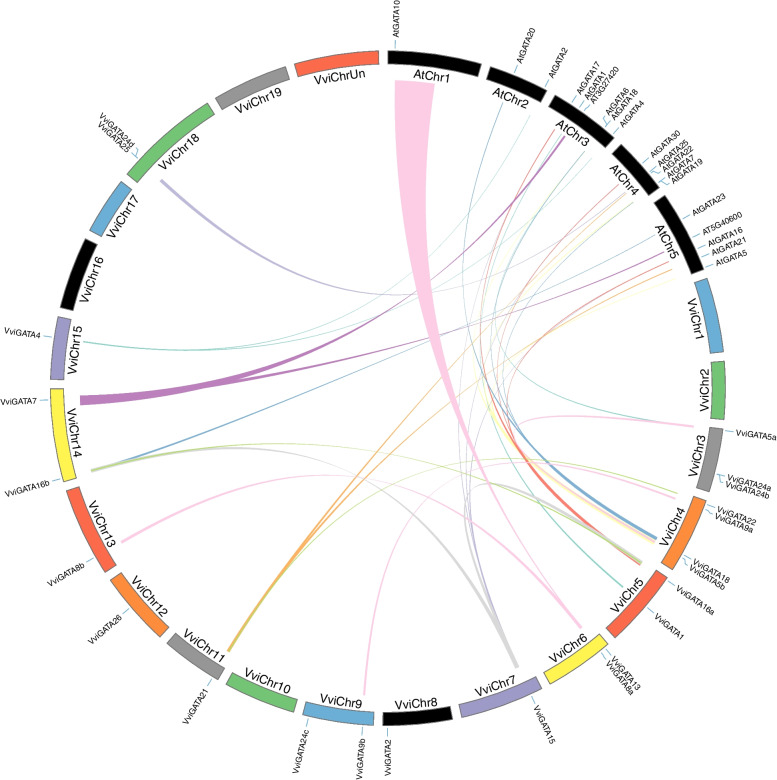
Chromosomal location and synteny analysis of *GATA* genes between grapevine and *Arabidopsis thaliana*, or grapevine alone. The chromosome number is shown at the bottom of each chromosome. The colored lines represent segmental duplication events between grapevine and *A. thaliana*, or grapevine alone

According to the grapevine reference genome VCost.v3 annotation [30], the 23 *VviGATA* genes are unevenly distributed among the thirteen chromosomes (Fig. 3), potentially reflecting segmental and tandem duplication, which are key driving forces in the evolution of large gene families [31]. Seven *VviGATA* gene pairs showed evidence of segmental duplication events: *VviGATA5a* to *VviGATA5b*, *VviGATA8a* to *VviGATA8b*, *VviGATA9a* to *VviGATA9b*, *VviGATA15* to *VviGATA16a*, *VviGATA15* to *VviGATA16b*, *VviGATA16a* to *VviGATA16b* and *VviGATA21* to *VviGATA22*. Only one pair (*VviGATA24d* to *VviGATA25* on chromosome 18) showed evidence of tandem duplication (Fig. 3, Additional file 6: Table S4), and both these genes were Group III members (Fig. 2A).

Next, the synteny of GATA gene pairs between the genomes of grapevine and *A. thaliana* was investigated and 23 orthologous gene pairs, comprising 12 *VviGATA* genes and 17 *AtGATA* genes, were identified. Of these, four orthologous pairs were determined to be single grapevine-to-*A. thaliana* pairs, while some *VviGATA* genes had multiple orthologous pairs in *A. thaliana*; *VviGATA5b* for example, had syntenic relationships with *AtGATA5*, *AtGATA6* and *AtGATA7* (Fig. 3, Additional file 7: Table S5). We note that AT3G27420 and AT5G40600 were not included in the *A. thaliana* GATA family, even though all contained a BET domain which was also found in VviGATA7 (Additional file 4: Fig. S2). We identified three orthologous pairs where multiple grapevine genes corresponded to a single *A. thaliana* gene (Fig. 3, Additional file 7: Table S5), suggesting a specific example of expansion of the grapevine GATA family.

To investigate potential selective pressure for GATA pairs, we calculated the nonsynonymous (Ka) and synonymous (Ks) substitution rates. Since the Ka/Ks values of all GATA pairs < 1.00, they likely evolved under intense purifying selection. The divergence time of the synteny or tandem duplication events was estimated as between 93.57 and 184.70 million years ago (Mya) in grapevine alone, and between 82.35 to 363.12 Mya between grapevine and *A. thaliana* (Additional file 6: Table S4, Additional file 7: Table S5).

### Analysis of *cis*-acting regulatory elements in the promoters of *VviGATA* genes

**Fig. 4:**
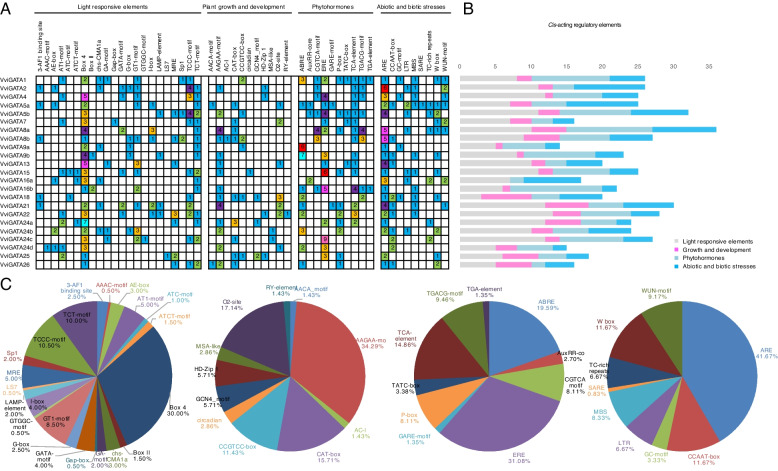
Analysis of *cis*-acting regulatory elements in *VviGATA* promoters. **A** Category (light responsive elements, plant growth and development, phytohormones, abiotic and biotic stresses) and the numbers of *cis*-acting regulatory elements in different members of the *VviGATA* gene family. **B** The sum of the *cis*-acting regulatory elements in each category. **C** The relative proportions of each *cis*-acting regulatory element in each category

To investigate the potential transcriptional regulation of *VviGATA* genes, we searched for putative *cis*-acting regulatory elements in their promoter regions (Additional file 1: Table S1). Four categories were identified, with light responsiveness accounting for the largest proportion (37%), as well as growth and development, phytohormones and biotic and abiotic stress (Fig. 4). The light responsive category contained Box 4, TCT-motif, MRE, GATA-motif, I-box and G-box. Among them, Box 4 (30%) was present in the promoter regions of all the *VviGATA* genes other than *VviGATA2* and *VviGATA18*. Additionally, *cis*-acting regulatory elements associated with growth and development (O2-site for zein metabolism regulation, CAT-box for meristem expression, HD-Zip 1 for differentiation of the palisade mesophyll cells, GCN4_motif for endosperm expression, MSA-like for cell cycle regulation, Circadian for circadian control) and hormone response (ERE for ethylene, ABRE for abscisic acid, TCA-element for salicylic acid, TGACG-motif for MeJA, P-box for gibberellin, AuxRR-core for auxin) were also identified. Various stress-related elements, including ARE, W box, CCAAT-box, WUN-motif, MBS, TC-rich repeats and LTR were identified in the promoter regions of all *VviGATA* genes. Of these, 22 had at least one stress-responsive motif. Lastly, an RY-element, annotated as associated with seed-specific regulation, was found in the *VviGATA22* promoter (Fig. 4).

### *VviGATA* expression patterns in grapevine tissues and fruit developmental stages

**Fig. 5:**
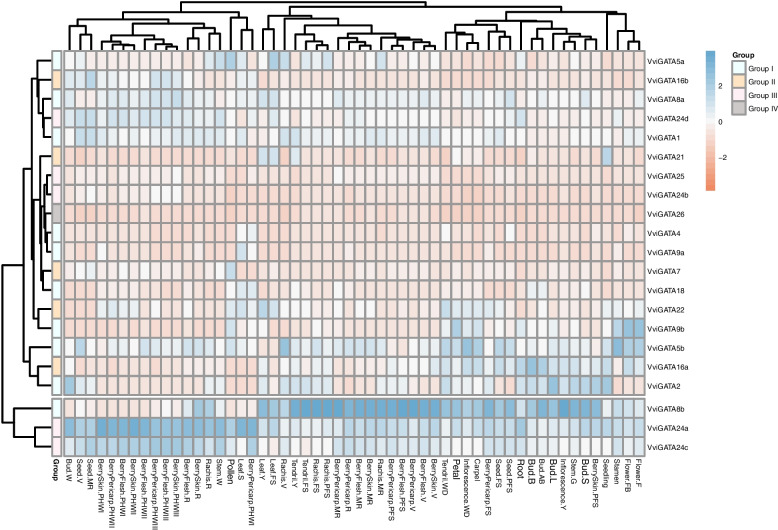
*VviGATA* expression profiles in various tissues at different developmental stages. *VviGATA* transcript levels in various tissues were investigated based on the mean expression value of each gene in a public transcriptome database [32]. The cyan and orange colors represent the higher and lower relative expression levels, respectively. Bud (-L: latent bud, -W: winter bud, -S: bud swell, -B: bud burst, -AB: after-burst); Inflorescence (-Y: young inflorescence, -WD: well developed inflorescence); Flower (-FB: flowering begins, -F: flowering); Tendril (-Y: young tendril, -WD: well developed tendril, -FS: mature tendril); Leaf (-Y: young leaf, -FS: mature leaf, -S: senescencing leaf); Berry Pericarp (-FS: fruit set, -PFS: post-fruit set, -V: véraison, -MR: mid-ripening, -R: ripening, -PHWI: post-harvest withering I, -PHWII: post-harvest withering II, -PHWIII: post-harvest withering III); Berry Skin/Flesh (-PFS: post-fruit set, -V: véraison, -MR: mid-ripening, -R: ripening, -PHWI: post-harvest withering I, -PHWII: post-harvest withering II, -PHWIII: post-harvest withering III); Seed (-FS: fruit set, -PFS: post-fruit set, -V: véraison, -MR: mid-ripening); Rachis (-FS: fruit set, -PFS: post-fruit set, -V: véraison, -MR: mid-ripening, -R: ripening); Stem (-G: green stem, -W: woody stem)

The expression atlas of all the *VviGATA* genes was created using microarray data from 54 combinations of organs/tissues at different developmental stages [32]. This showed that only a small subset had similar expression profiles in all organs/tissues. For example, *VviGATA8b*, *VviGATA24a* and *VviGATA24c* were highly expressed and relatively ubiquitously, whereas *VviGATA4*, *VviGATA24b*, *VviGATA25* and *VviGATA26* were expressed at very low levels in nearly all organs/tissues (Fig. 5). Other genes showed tissue-specific expression, indicative of functional diversification, such as *VviGATA7* and *VviGATA9a*, which were only expressed in pollen and senescing leaves.

**Fig. 6:**
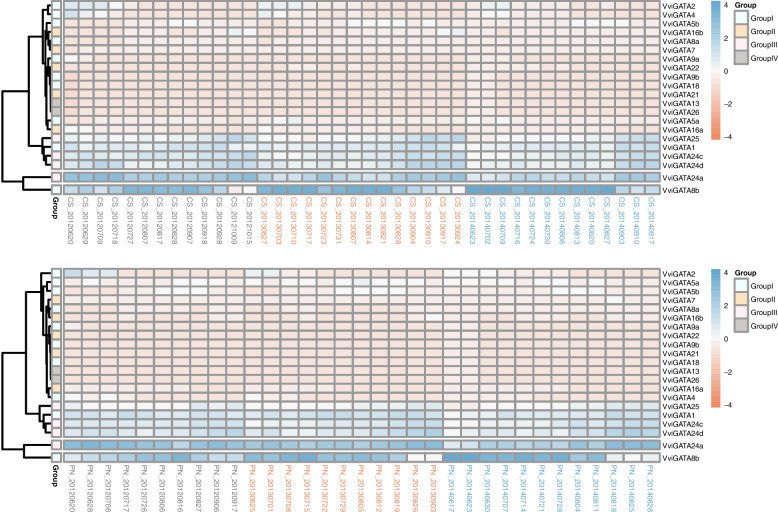
*VviGATA*s expression patterns during development and berry ripening in grapevine. *VviGATA* transcript levels during development and berry ripening were investigated based on the mean expression value of each gene in a public transcriptome database [33]. The samples were collected every week from fruit set to maturity in two grapevine genotypes (*Vitis. vinifera* cv ‘Cabernet Sauvignon’ and *V. vinifera* cv ‘Pinot Noir’) for three consecutive years (2012, 2013 and 2014)

To gain insights into the putative roles of *VviGATA* genes during berry development and ripening, we used RNA sequencing datasets from the Gene Expression Omnibus (GEO) database [33]. As shown in Fig. 6, the expression trends for individual genes were mostly consistent between three consecutive years (2012, 2013 and 2014) from fruit set to maturity and in both ‘Cabernet Sauvignon’ and ‘Pinot Noir’. *VviGATA1*, *VviGATA24a, VviGATA24c*, *VviGATA24d* and *VviGATA25* were more highly expressed in immature than mature berries in the two genotypes, whereas *VviGATA8b* showed the opposite pattern. We noted that *VviGATA2* was only highly expressed at fruit set, suggesting that it might not be involved in a regulatory switch during grapevine berry development.

### *VviGATA* expression patterns in response to abiotic stresses

**Fig. 7:**
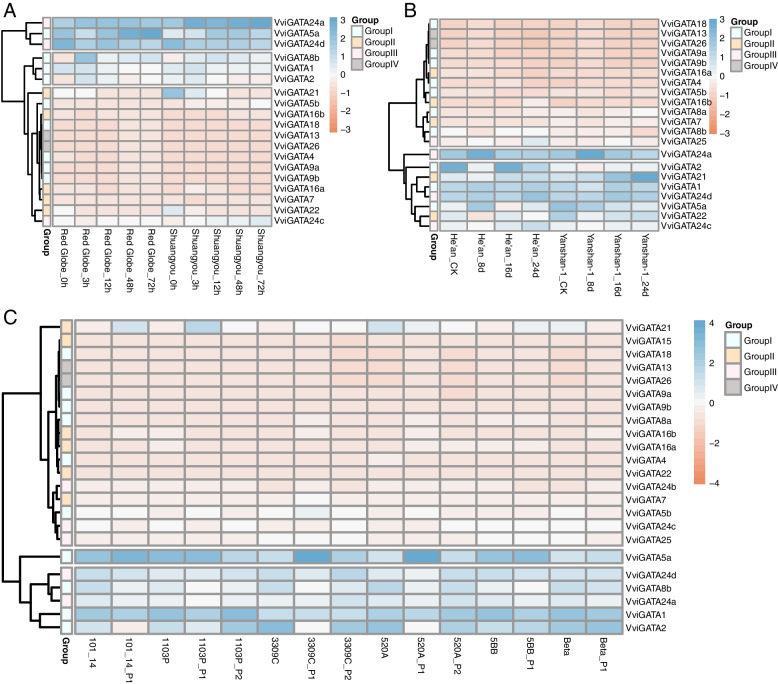
*VviGATA* expression analysis in response to various abiotic stresses including cold (**A**), drought (**B**) and salt (**C**) treatments. Data used in the analysis were collected from the grapevine public transcriptome database [34–36]

We further analyzed *VviGATA* expression patterns following exposure to different abiotic stress treatments, including cold, drought and salt stresses, using published grapevine transcriptome data [34–36]. Several *VviGATA* genes were strongly up-regulated, such as *VviGATA1*, *VviGATA5a* and *VviGATA24a* following drought, salt and cold treatments, respectively. In contrast, other *VviGATA* genes showed opposite expression patterns under different abiotic stress. For example, *VviGATA21* responded to all treatments, but displayed down-regulation following cold stress, whereas up-regulation during other abiotic stresses. Notably, *VviGATA24d* was significantly induced by all abiotic stresses tested (Fig. 7). To support the reads per kilobase per million mapped reads (RPKM) results in the transcriptome, the expression level of *VviGATAs* in response to cold treatment (Fig. 7) was determined using Real-Time Quantitative PCR (RT-qPCR), and results of both analysis approaches were generally consistent (Additional file 8: Fig. S3).

### Subcellular localization and transcriptional activity of five GATA proteins

**Fig. 8:**
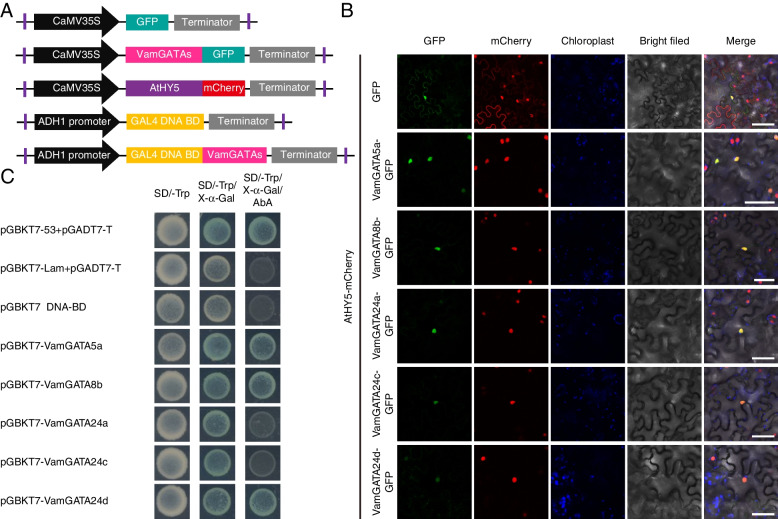
The subcellular localization and transcriptional activity analysis of five *GATA* genes. **A** Illustration of the constructs used for the subcellular localization and transcriptional activity assays. **B** Subcellular localization of five GATA proteins. The 35S-VamGATAs-GFP and 35S-GFP control were transiently expressed in *Nicotiana benthamiana* leaf epidermal cells. The *Arabidopsis thaliana* 35S-AtHY5-mCherry was chosen as a nuclear localization marker gene [37]. Scale bar = 40 μm. **C** Transactivation activity assay of five GATA proteins in yeast. The transformed yeast cells were grown on SD/-Trp/X-α-gal/AbA medium and blue color indicate transcriptional activity. pGBKT7-53 co-transformed with pGADT7-T was used as the positive control, and pGBKT7-Lam co-transformed with pGADT7-T was used as the negative control

To obtain evidence in support of the predicted localization pattern of VviGATA proteins in cells, five genes, which strongly responsed to various abiotic stresses including cold, drought and salt treatments (Fig. 7), were cloned from cold-resistant *V. amurensis* ‘Shuangyou’ for transient expression as fusion proteins with a green fluorescent protein (GFP) reporter in *Nicotiana benthamiana* leaf epidermal cells (Additional file 9: Fig. S4). The *A. thaliana* AtHY5 (ELONGATED HYPOCOTYL 5; AT5G11260.1) was chosen as a nuclear localization marker gene [37] to co-transform with VamGATAs. The GFP signals from VamGATA5a-GFP, VamGATA8b-GFP, VamGATA24a-GFP, VamGATA24c-GFP and VamGATA24d-GFP fusion proteins were all seen to overlap with the AtHY5-mCherry signals in the nucleus (Fig. 8A, B), which was consistent with the predicted results except for VamGATA24a (Table 1). Additionally, the transcriptional activation activities of the five VamGATA proteins were analyzed using a yeast two-hybrid system. Positive blue colonies of yeast cells transformed with pGBKT7-VamGATA5a, pGBKT7-VamGATA8b and pGBKT7-VamGATA24d were observed on a selective solid medium plate lacking tryptophan, and supplemented with 5-Bromo-4-chloro-3-indolyl-α-D-galactopyranoside and Aureobasidin A (SD/-Trp/X-α-Gal/AbA), while yeast cells transformed with pGBKT7-VamGATA24a and pGBKT7-VamGATA24c did not survive, suggesting that VamGATA5a, VamGATA8b and VamGATA24d had transcriptional activity, while VamGATA24a and VamGATA24c had no such activity (Fig. 8A, C).

## Discussion

In this investigation, 23 *VviGATA* genes were identified; the same number as in *Eucalyptus grandis* [38] and similar to *O. sativa* (28), *A. thaliana* (30), *S. lycopersicum* (30) and *P. edulis* (31) [3, 8, 39], but fewer than *M. domestica* (35), *A. hypogaea* (45), *S. tuberosum* (49), *T. aestivum* (79) and *Brassica napus* (96) [6, 9–11, 40]. The genes were named based on the current nomenclature [29] and their detailed information is listed in Table 1, Additional file 1: Table S1 and Additional file 2: Table S2. As in other plant species, such as *A. thaliana* and *M. domestica* [3, 6], we found that in grapevine Clade I was the largest (Fig. 1). The division into clades was the same whether the grapevine genes were analyzed alone or with genes from other species (Figs. 1 and 2A), which has also been shown for the *T. aestivum* GATA gene family [9].

The conserved domains (Additional file 3: Fig. S1) were mostly consistent with those previously identified in *A. thaliana* [3] and the variation seen in this study has also been observed in other species. For instance, *B. napus* BnGATA2.8 and BnGATA2.26 contain N-X_2_-C-X_18_-C-X_2_-C, and *Cucumis sativus* Csa4G286370 has two extra amino acids forming a C-X_4_-C-X_18_-C-X_2_-C domain [40, 41]. In *A. thaliana*, many GATA proteins with CCT, TIFY and BET domains have a role in integrating day length and rhythmicity, regulation of seedlings with elongated hypocotyls and petioles, and embryogenesis [42–44]. We found that five VviGATA proteins from Group III and VviGATA7 from Group IV also contained these domains (Fig. 2A, Additional file 4: Fig. S2), and speculate that they may have similar functions in grapevine. As expected, most of the closely related members from the same groups had common motif compositions and exon–intron structures (Fig. 2, Additional file 5: Table S3). Indeed, we observed five gene pairs (*VviGATA*5a/*VviGATA*5b, *VviGATA*8a/*VviGATA*8b, *VviGATA*15/*VviGATA*16a, *VviGATA*15/*VviGATA*16b and *VviGATA*16a/*VviGATA*16b) with the same number of exons and motifs, suggesting that they might have been involved in tandem or segmental duplication events, which was supported by our synteny analysis (Figs. 2 and 3, Additional file 6: Table S4). The conserved motif 2 was only found in the grapevine GATA Group I (Fig. 2B, Additional file 5: Table S3), indicating unique functions for these genes, but further evidence is needed to verify this. Moreover, the exon number in the grapevine genes varied from 1 to 18 (Fig. 2C), which is distinct from that in *A. thaliana* (2 to 8) and rice (2 to 9) [3]. This suggests that the *VviGATA* genes have undergone moderate structure divergence over the course of evolution.

As shown in Fig. 3, the 23 *VviGATA* genes are unevenly distributed on the grapevine chromosomes, which may be reflect the differences in the size and structure of the chromosomes. We found seven segmental duplications and only one tandem duplication (Fig. 3, Additional file 6: Table S4), indicating that the grapevine GATA genes have not undergone large scale gene expansion, which is similar to *C. sativus* [41]. The 23 orthologous GATA gene pairs involved in segmental duplications between grapevine and *A. thaliana* represent more than half of the GATA genes from each species. For example, *VviGATA21* showed syntenic relationship with *AtGATA21*/*AtGNC* and *AtGATA22/AtGNL/AtCGA1* (Fig. 3, Additional file 7: Table S5). *A. thaliana* GNC and GNL/CGA1 directly repress the transcription of *SUPPRESSOR OF OVEREXPRESSION OF CONSTANS1* (*SOC1*). Conversely, SOC1 represses the transcription of *GNC* and *GNL/CGA1* to control greening and cold tolerance [45]. In this study, we found that *VviGATA21* was expressed at relatively high levels during cold stress in cold-resistant *V. amurensis* ‘Shuangyou’ (Fig. 7, Additional file 8: Fig. S3), also implicating it in abiotic stress responses in grapevine.

The development and ripening of grapevine berries directly affect the quality of fresh fruit and vinification, and our results revealed that some *VviGATA* genes were highly expressed in leaves, berries and flowers (Fig. 5), implying potential roles in development and berry ripening. There are previously reported examples of *GATA* genes being involved in these processes in *A. thaliana*, where GATA proteins have been found to be involved in chlorophyll synthesis and floral development [46, 47] and *Chrysanthemum morifolium*, where CmGATA4 acts as a negative regulator to lower the expression of *CmCCD4a-5* resulting in carotenoid accumulation in the mutant [48]. Here, *VviGATA24a* and *VviGATA24c*, which are closely related members of Group III, both showed high expression levels in berries (Figs. 2 and 5), and RNA-seq data also showed that they are highly expressed from fruit set to maturity (Fig. 6). Furthermore, many *cis*-acting regulatory elements related to light responses, such as Box 4 and TCT-motif, were identified in the *VviGATA24a* and *VviGATA24c* promoters (Fig. 4), consistent with functions in grapevine growth and development.

Previous studies have identified plant GATA genes that are involved in responses to drought, salt and cold stresses [19, 20, 45]. For instance, *PdGNC* from *P. deltoides* was found to confer drought tolerance by mediating stomatal closure [49], and *SlGATA17* was reported to negatively modulate salinity tolerance in *S. lycopersicum* [50]. In addition, PpGATA12 from *Prunus persica* was observed to respond to low temperature and brassinosteroid signaling and to induced the transcription of sucrose and energy metabolism-related genes to enhance fruit tolerance to cold stress [51]. We found that *VviGATA5a* contains LTR elements in the promoter involved in low temperature responsiveness, consistent with the RNA-seq expression data (Figs. 4 and 7), and indicating its potential function in cold stress responses. The segmentally duplicated genes *VviGATA21* and *VviGATA22*, were strongly upregulated by drought treatment (Figs. 3 and 7), and might positively regulate drought responses. In addition, three *VviGATAs* (*VviGATA5a*, *VviGATA24a* and *VviGATA24d*) were upregulated in cold, drought and salt treatments (Fig. 7), suggesting that these three genes may integrate different stress signals.

In this study, subcellular localization software predicted that approximately 87% GATA proteins were located in the nucleus (Table 1). And all five tested *VamGATAs* from *V. amurensis* ‘Shuangyou’ were found to be located in the nucleus (Fig. 8B), which is consistent with the localization of most TFs, and similarly to IbGATA24 from sweet potato that is associated with drought and salt stress tolerance [20]. Interestingly, the VviGATA24a was a predicted chloroplast protein (Table 1). The reason might be that they are different genetic backgrounds between *V. vinifera* ‘Pinot Noir’ (the grapevine reference genome) and *V. amurensis* ‘Shuangyou’. Notably, VamGATA24a and VamGATA24c did not show any transactivation activation ability (Fig. 8C) and we suggest that they may require post-translational modification or interaction with other proteins to regulate downstream target genes.

## Conclusions

In the present study, 23 *VviGATA* genes were identified from the latest annotated version of *V. vinifera* genome. These genes were divided into four groups based on phylogeny, which was further supported by highly similar conserved motif compositions and exon–intron configurations. Segmental and tandem duplication events were found to have contributed to the expansion of the grapevine GATA gene family. Numerous *cis*-acting regulatory elements and expression analysis indicated that VviGATA proteins might participate in growth and development, as well as abiotic stresses. Additionally, the subcellular location and transactivation ability of five GATAs was verified, suggesting that GATA proteins might activate the expression of downstream target genes in the nucleus. Taken together, these findings provide a foundation for further research into the functions of *GATA* genes in grapevine.

## Methods

### Identification and annotation of *GATA* genes in the grapevine genome

A HMM profile of the GATA domain (PF00320), downloaded from Pfam (https://www.ebi.ac.uk/interpro/) [52], was used to identify the potential GATA members in the grapevine reference genome assembly (12X.v2) VCost.v3 annotation [30, 53], using HMMER3.0 software [54] with E-values < 1e^−5^. The presence of the GATA domain in all putative proteins was then manually confirmed using the SMART (http://smart.embl-heidelberg.de) [55] and Conserved Domain Databases (CDD) (https://www.ncbi.nlm.nih.gov/Structure/cdd/wrpsb.cgi) [56]. A range of GATA protein properties, including molecular weight, isoelectric points, instability index, aliphatic index and GRAVY, were determined using the ExPASy ProtParam tool (http://web.expasy.org/protparam/) [57], and protein subcellular localizations were predicted using WoLF PSORT (https://wolfpsort.hgc.jp) [58].

### Conserved domain alignments and phylogenetic analysis

Multiple sequence alignments of the conserved GATA domain were performed using DNAMAN (Version 7.0.2, Lynnon Biosoft), and sequence logos were created using Weblogo 3 (http://weblogo.threeplusone.com) [59]. For full length protein sequence alignments, the muscle method in the MEGA 11 software package [60] was used, and phylogenetic trees were constructed with the Neighbor-Joining approach, with 1,000 bootstrap replications, and the following parameters: p-distance model, uniform rates, same (homogeneous) pattern, and pairwise deletion gaps. The GATA protein sequences from *A. thaliana* (*AtGATA*) and rice (*O. sativa*) (*OsGATA*) [3, 4], apple (*M. domestica*) (*MdGATA*) [6], tomato (*S. lycopersicum*) (*SlGATA*) [8], bamboo (*P. edulis*) (*PeGATA*) [39] and potato (*S. tuberosum*) (*StGATA*) [11] were downloaded from the genome databases corresponding to each species.

### Chromosomal localization and synteny analysis

The chromosomal location of each *VviGATA* gene was identified using the physical location information from the VCost.v3 gene annotation [30, 53]. The synteny blocks of the grapevine *GATA* genes, as well as between grapevine and *A. thaliana* genes, were analyzed using MCScanX software [61], and globe plot diagrams were made using Circos-0.69–6 (http://circos.ca) [62]. The Ka and Ks substitution rates of each gene pair were calculated using TBtools [63]. The Ks values were used to calculate the divergence time with the following formula: T = Ks/2λ × 10^–6^ Mya (λ = 6.5 × 10^–9^ for grapevine) [64].

### Exon–intron structure, conserved motif and *cis*-acting regulatory element analysis

Exon and intron structures of the confirmed *GATA* genes were determined based on CDS and each full-length sequence in the grapevine reference genome assembly (12X.v2) and its VCost.v3 annotation [30, 53]. The exon–intron diagrams were generated using Gene Structure Display Server 2.0 (http://gsds.cbi.pku.edu.cn) [65]. The conserved motifs of the GATA proteins identified using the MEME analysis tool (http://meme-suite.org/tools/meme) [66] with a limitation of 13 motifs and default parameters. Only motifs with E-value < 0.05 were present. TBtools [63] was used to generate a map of the conserved motifs. The promoter sequences (defined as 2,000 bp upstream from each ATG start codon) of the *VviGATA* genes were obtained from the grapevine reference genome [53] and submitted to the PlantCARE database (http://bioinformatics.psb.ugent.be/webtools/plantcare/html/) [67] to identify *cis-*acting regulatory elements.

### *VviGATA* expression profiles in various organs and different berry developmental stages

*VviGATA* (*V. vinifera* cv. ‘Corvina’) microarray expression data from different vegetative and reproductive organs at various developmental stages were acquired from the GEO datasets from the GSE36128 series [32]. *VviGATA* expression patterns in samples collected every week from fruit set to maturity in two grapevine genotypes (*V. vinifera* cv. ‘Cabernet Sauvignon’ and *V. vinifera* cv. ‘Pinot Noir’) for three consecutive years (2012, 2013 and 2014) were obtained from the GEO datasets from the GSE98923 series [33].

### Expression patterns in response to different abiotic stress conditions

*VviGATA* RNA-seq data reflecting responses to cold, drought and salt stress were retrieved from published datasets, as follows: the leaves of one-year-old potted grapevine plants of cold-resistant *V. amurensis* ‘Shuangyou’ and cold-sensitive *V. vinifera* cv. ‘Red Globe’ after 0°C treatment for 3, 12, 48, and 72 h [35]. Leaves of two-year-old potted cutting seedlings of the drought-resistant Chinese wild *V. yeshanensis* accession Yanshan-1 and the drought-sensitive *V. riparia* accession He’an after drought stress for 0, 8, 16, and 24 d [34]; six two-year-old pot-grown grapevine rootstocks, including salt-tolerant varieties 3309C (*V. riparia* × *V. rupestris*), 520A (*V. berlandieri* × *V. riparia*) and 1103P (*V. berlandieri* × *V. rupestris*) and the intolerant varieties 5BB (*V. berlandieri* × *V. riparia*), 101–14 (*V. riparia* × *V. rupestris*) and Beta (*V. riparia* × *V. labrusca*) watered for 2 consecutive days with 130 mmol L^−1^ NaCl solution to induce salinity stress [36].

The RPKM values were used to assess *VviGATA* expression and all heatmaps were generated using the R version 4.2.2 software package (https://www.r-project.org/).

### Plant materials, RNA isolation and RT-qPCR

*V. amurensis* ‘Shuangyou’ samples were obtained from the grapevine germplasm resource orchard of Northwest A&F University, Yangling, Shaanxi, China (34°20′N, 108°24′E). Leaves were collected and immediately frozen in liquid nitrogen and stored at -80°C until further use. Total RNA was collected using an EZNA Plant RNA Kit (Omega Bio-tek, Norcross, GA, USA). First-strand cDNA was obtained by reverse transcription of 1 μg DNA-free total RNA using a Prime Script RT reagent Kit (TaKaRa Biotechnology, Dalian, China), following the manufacturer’s instructions. The full-length CDS of five *VamGATA* genes were amplified with the high fidelity PrimeSTAR^®^ Max DNA Polymerase (TaKaRa Biotechnology, Dalian, China), according to the manufacturer’s instructions.

RT-qPCR analysis was performed using the ChamQ SYBR Color qPCR Master Mix (Vazyme, Nanjing, China) with the following parameters: 95°C for 30 s, 40 cycles at 95°C for 5 s, and 60°C for 30 s. Relative expression levels were calculated using the 2^−ΔΔCT^ method [68] with the grapevine *ACTIN1* (Vitvi04g01613.t01) as a reference gene. Primers were designed using Primer Premier 5.0 software (PREMIER Biosoft International, Palo Alto, CA, USA) and listed in Additional file 10: Table S6.

Significant differences were analyzed using one-way ANOVA, followed by Fisher's least significant difference method (*p* < 0.05) with SPSS Version 25 software (SPSS, Inc., Chicago, IL, USA). Graphics were drawn using GraphPad Prism Version 9.1.1 software (GraphPad, Inc., San Diego, CA, USA).

### Subcellular localization and transcriptional activity of GATA proteins

The CDSs of *VamGATA* genes from *V. amurensis* ‘Shuangyou’ without stop codons were inserted with *Kpn* I and *BamH* I (Takara Biomedical Technology, Beijing, China) into the pCAMBIA2300-GFP vector (CAMBIA, Canberra, Australia) driven by CaMV35S using the ClonExpress II One Step Cloning Kit (Vazyme, Nanjing, China) to produce 35S-VamGATA-GFP recombinant expression vectors. The *A. thaliana* nuclear protein AtHY5 combined with mCherry (35S-AtHY5-mCherry) were used as marker genes [37]. These vectors were then co-transformed into *Agrobacterium tumefaciens* GV3101 (pSoup-p19) and infiltrated into the leaves of *N. benthamiana* as previously described [69]. GFP and mCherry signals were detected using a confocal laser scanning microscope (LEICA TCS SP8, Germany) with excitation wavelengths of 488 nm and 552 nm, respectively.

The full-length *VamGATA* CDSs were cloned into the pGBKT7 vector, and the resulting plasmids were transformed into the Y2HGold yeast strain according to the Yeastmaker™ Yeast Transformation System 2 User Manual (Clontech Laboratories, Mountain View, CA, USA). Transcriptional activation activity was indicated by the presence of blue colonies growing on a selective solid medium plate lacking tryptophan, and supplemented with 40 μg mL^−1^ X-α-Gal and 200 ng mL^−1^ AbA. pGBKT7-53 co-transformed with pGADT7-T was used as the positive control, and pGBKT7-Lam co-transformed with pGADT7-T was used as a negative control. Primers are listed in Additional file 10: Table S6.

### Supplementary Information

**Additional file 1: Table S1.** The coding sequence and promoter sequences of grapevine* GATA* family members.

**Additional file 2: Table S2.** The protein sequences of GATA family members from *Vitis vinifera*, *Arabidopsis thaliana*, *Oryza sativa*, *Malus domestica*, *Solanum lycopersicum*, *Phyllostachys edulis* and *Solanum tuberosum*.

**Additional file 3: Fig. S1.** Alignment of conserved GATA domains from the 23 VviGATA transcription factors.

**Additional file 4: Fig. S2. **Distribution of conserved GATA domains in VviGATA proteins.

**Additional file 5: Table S3.** GATA protein motif sequences identified using the MEME tool.

**Additional file 6: Table S4. **Segmental and tandem duplications within the grapevine *VviGATA *gene family and Ka/Ks ratio analysis of segmental and tandem duplicated gene pairs.

**Additional file 7: Table S5. **Segmental duplications of *GATA *genes between grapevine and *Arabidopsis* and Ka/Ks ratio analysis of segmentally duplicated gene pairs.

**Additional file 8: Fig. S2. **Alignment of the coding sequences of five cloned* GATA *genes form *Vitis amurensis* ‘Shuangyou’.

**Additional file 9: Fig. S3. **Real-Time Quantitative PCR analysis of expression of selected *VviGATA* genes. The grapevine *ACTIN1* gene was used as an internal control to normalize expression levels. Letters indicate significance differences in gene expression using one-way ANOVA, followed by Fisher's least significant difference method (*p* < 0.05).

**Additional file 10: Table S6. **Primers used for cloning, subcellular localization, transcriptional activity and Real-Time Quantitative PCR studies.

## Data Availability

The grapevine reference genome assembly (12X.v2) and its VCost.v3 gene annotation downloaded from URIG website (https://urgi.versailles.inra.fr/Species/Vitis/Annotations) [30]. The microarray data for expression profiles in in various organs and different berry developmental stages are available on NCBI GEO under the accession number GSE36128 (https://www.ncbi.nlm.nih.gov/geo/query/acc.cgi?acc=GSE36128) [32] and GSE98923 (https://www.ncbi.nlm.nih.gov/geo/query/acc.cgi) [33], respectively. RNA-Seq data in response to different abiotic stress conditions were retrieved from the published supplementary data sets [34–36]. The datasets supporting the results of this article are included in the article and Additional files.

## Abbreviations

TFs: Transcription factors
HMM: Hidden Markov Model
CDS: Coding sequence
GRAVY: Grand average of hydropathicity
BET: Bromodomain and extra-terminal
Ka: Nonsynonymous
Ks: Synonymous
Mya: Million years ago
GEO: Gene Expression Omnibus
GFP: Green fluorescent protein
SD/-Trp: Lacking tryptophan
X-α-Gal: 5-Bromo-4-chloro-3-indolyl-α-D-galactopyranoside
AbA: Aureobasidin A
CDD: Conserved Domain Databases
RPKM: Reads per kilobase per million mapped reads
RT-qPCR: Real-Time Quantitative PCR

## References

[1] Tian F, Yang DC, Meng YQ, Jin J, Gao G (2020). PlantRegMap: charting functional regulatory maps in plants. Nucleic Acids Res.

[2] Lowry JA, Atchley WR (2000). Molecular evolution of the GATA family of transcription factors: conservation within the DNA-binding domain. J Mol Evol.

[3] Reyes JC, Muro-Pastor MI, Florencio FJ (2004). The GATA family of transcription factors in Arabidopsis and rice. Plant Physiol.

[4] Schwechheimer C, Schroder PM, Blaby-Haas CE (2022). Plant GATA Factors: Their Biology, Phylogeny, and Phylogenomics. Annu Rev Plant Biol.

[5] Scazzocchio C (2000). The fungal GATA factors. Curr Opin Microbiol.

[6] Chen H, Shao H, Li K, Zhang D, Fan S, Li Y, Han M. Genome-wide identification, evolution, and expression analysis of GATA transcription factors in apple (*Malus* x *domestica* Borkh.). Gene*.* 2017; 627:460–72. 10.1016/j.gene.2017.06.049 28669931

[7] Daniel-Vedele F, Caboche M (1993). A tobacco cDNA clone encoding a GATA-1 zinc finger protein homologous to regulators of nitrogen metabolism in fungi. Mol Gen Genet.

[8] Yuan Q, Zhang C, Zhao T, Yao M, Xu X (2018). A Genome-Wide Analysis of GATA Transcription Factor Family in Tomato and Analysis of Expression Patterns. Int J Agric Biol.

[9] Feng X, Yu Q, Zeng J, He X, Liu W (2022). Genome-wide identification and characterization of GATA family genes in wheat. BMC Plant Biol.

[10] Li X, Deng X, Han S, Zhang X, Dai T (2023). Genome-Wide Identification and Expression Analysis of GATA Gene Family under Different Nitrogen Levels in *Arachis hypogaea* L. Agronomy.

[11] Yu R, Chang Y, Chen H, Feng J, Wang H, Tian T, Song Y, Gao G. Genome-wide identification of the GATA gene family in potato (*Solanum tuberosum* L.) and expression analysis. J Plant Biochem Biot*.* 2021; 31(1):37–48.

[12] Luo XM, Lin WH, Zhu S, Zhu JY, Sun Y, Fan XY, Cheng M, Hao Y, Oh E, Tian M, Liu L, Zhang M, Xie Q, Chong K, Wang ZY (2010). Integration of light- and brassinosteroid-signaling pathways by a GATA transcription factor in *Arabidopsis*. Dev Cell.

[13] Richter R, Behringer C, Zourelidou M, Schwechheimer C (2013). Convergence of auxin and gibberellin signaling on the regulation of the GATA transcription factors *GNC* and *GNL* in Arabidopsis thaliana. Proc Natl Acad Sci U S A.

[14] An Y, Zhou Y, Han X, Shen C, Wang S, Liu C, Yin W, Xia X (2020). The GATA transcription factor GNC plays an important role in photosynthesis and growth in poplar. J Exp Bot.

[15] Wei X, Li Y, Zhu X, Liu X, Ye X, Zhou M, Zhang Z (2023). The GATA transcription factor TaGATA1 recruits demethylase TaELF6-A1 and enhances seed dormancy in wheat by directly regulating *TaABI5*. J Integr Plant Biol.

[16] Liu X, Zhu X, Wei X, Lu C, Shen F, Zhang X, Zhang Z (2020). The wheat LLM-domain-containing transcription factor TaGATA1 positively modulates host immune response to *Rhizoctonia cerealis*. J Exp Bot.

[17] Zhang YJ, Zhang Y, Zhang LL, Huang HY, Yang BJ, Luan S, Xue HW, Lin WH (2018). *OsGATA7* modulates brassinosteroids-mediated growth regulation and influences architecture and grain shape. Plant Biotechnol J.

[18] Zhang YJ, Zhang Y, Zhang LL, He JX, Xue HW, Wang JW, Lin WH (2022). The transcription factor OsGATA6 regulates rice heading date and grain number per panicle. J Exp Bot.

[19] Zhang H, Wu T, Li Z, Huang K, Kim NE, Ma Z, Kwon SW, Jiang W, Du X (2021). OsGATA16, a GATA Transcription Factor, Confers Cold Tolerance by Repressing *OsWRKY45-1* at the Seedling Stage in Rice. Rice.

[20] Zhu H, Zhai H, He S, Zhang H, Gao S, Liu Q (2022). A novel sweetpotato GATA transcription factor, IbGATA24, interacting with IbCOP9-5a positively regulates drought and salt tolerance. Environ Exp Bot.

[21] Yu YH, Bian L, Yu KK, Yang SD, Zhang GH, Guo DL (2020). Grape (*Vitis davidii*) *VdGATA2* functions as a transcription activator and enhances powdery mildew resistance via the active oxygen species pathway. Sci Hortic.

[22] Zhang X, Wu Y, Li Z, Song C, Wang X. Advancements in plant regeneration and genetic transformation of grapevine (*Vitis* spp.). J Integr Agric*.* 2021; 20(6):1407–34.

[23] Dong Y, Duan S, Xia Q, Liang Z, Dong X, Margaryan K, Musayev M, Goryslavets S, Zdunić G, Bert PF, Lacombe T, Maul E, Nick P, Bitskinashvili K, Bisztray GD, Drori E, De Lorenzis G, Cunha J, Popescu CF, Arroyo-Garcia R, Arnold C, Ergül A, Zhu Y, Ma C, Wang S, Liu S, Tang L, Wang C, Li D, Pan Y, Li J, Yang L, Li X, Xiang G, Yang Z, Chen B, Dai Z, Wang Y, Arakelyan A, Kuliyev V, Spotar G, Girollet N, Delrot S, Ollat N, This P, Marchal C, Sarah G, Laucou V, Bacilieri R, Röckel F, Guan P, Jung A, Riemann M, Ujmajuridze L, Zakalashvili T, Maghradze D, Höhn M, Jahnke G, Kiss E, Deák T, Rahimi O, Hübner S, Grassi F, Mercati F, Sunseri F, Eiras-Dias J, Dumitru AM, Carrasco D, Rodriguez-Izquierdo A, Muñoz G, Uysal T, Özer C, Kazan K, Xu M, Wang Y, Zhu S, Lu J, Zhao M, Wang L, Jiu S, Zhang Y, Sun L, Yang H, Weiss E, Wang S, Zhu Y, Li S, Sheng J, Chen W (2023). Dual domestications and origin of traits in grapevine evolution. Science.

[24] Wang Y, Liu Y, He P, Chen J, Lamikanra O, Lu J (1995). Evaluation of foliar resistance to *Uncinula Necato*r in *Chinese Wild Vitis* Species. Vitis.

[25] Liu L, Li H (2013). Review: Research progress in amur grape, *Vitis amurensis* Rupr. Can J Plant Sci.

[26] Huangfu C, Zhang H, Xiu J, Feng Y (1994). ‘Shuang You’ - a new hermaphrodite cultivar of *Vitis Amurensis* Rupr. Viticul Wine-making.

[27] Chen T, Peng J, Li M, Dou M, Lei Y, Wang Y, Xu Y (2022). Genetic Analysis of the Grapevine GATA Gene Family and Their Expression Profiles in Response to Hormone and Downy Mildew Infection. Horticulturae.

[28] Zhang Z, Ren C, Zou L, Wang Y, Li S, Liang Z (2018). Characterization of the GATA gene family in *Vitis vinifera*: genome-wide analysis, expression profiles, and involvement in light and phytohormone response. Genome.

[29] Grimplet J, Adam-Blondon AF, Bert PF, Bitz O, Cantu D, Davies C, Delrot S, Pezzotti M, Rombauts S, Cramer GR (2014). The grapevine gene nomenclature system. BMC Genomics.

[30] Canaguier A, Grimplet J, Di Gaspero G, Scalabrin S, Duchene E, Choisne N, Mohellibi N, Guichard C, Rombauts S, Le Clainche I, Berard A, Chauveau A, Bounon R, Rustenholz C, Morgante M, Le Paslier MC, Brunel D, Adam-Blondon AF. A new version of the grapevine reference genome assembly (12X.v2) and of its annotation (VCost.v3). Genomics Data*.* 2017; 14:56–62. 10.1016/j.gdata.2017.09.002 PMC5612791 28971018

[31] Cannon SB, Mitra A, Baumgarten A, Young ND, May G (2004). The roles of segmental and tandem gene duplication in the evolution of large gene families in *Arabidopsis thaliana*. BMC Plant Biol.

[32] Fasoli M, Dal Santo S, Zenoni S, Tornielli GB, Farina L, Zamboni A, Porceddu A, Venturini L, Bicego M, Murino V, Ferrarini A, Delledonne M, Pezzotti M (2012). The grapevine expression atlas reveals a deep transcriptome shift driving the entire plant into a maturation program. Plant Cell.

[33] Fasoli M, Richter CL, Zenoni S, Bertini E, Vitulo N, Dal Santo S, Dokoozlian N, Pezzotti M, Tornielli GB (2018). Timing and Order of the Molecular Events Marking the Onset of Berry Ripening in Grapevine. Plant Physiol.

[34] Cui X, Xue J, Zhang B, Chen C, Tang Y, Zhang P, Zhang J (2020). Physiological change and screening of differentially expressed genes of wild Chinese *Vitis yeshanensis* and American *Vitis riparia* in response to drought stress. Sci Hortic.

[35] Gu B, Zhang B, Ding L, Li P, Shen L, Zhang J (2020). Physiological Change and Transcriptome Analysis of Chinese Wild *Vitis amurensis* and *Vitis viniferain* Response to Cold Stress. Plant Mol Biol Rep.

[36] Zhao F, Zheng T, Liu Z, Fu W, Fang J (2022). Transcriptomic Analysis Elaborates the Resistance Mechanism of Grapevine Rootstocks against Salt Stress. Plants.

[37] Li QF, He JX (2016). BZR1 Interacts with HY5 to Mediate Brassinosteroid- and Light-Regulated Cotyledon Opening in *Arabidopsis* in Darkness. Mol Plant.

[38] Du K, Xia Y, Zhan D, Xu T, Lu T, Yang J, Kang X (2022). Genome-Wide Identification of the *Eucalyptus urophylla* GATA Gene Family and Its Diverse Roles in Chlorophyll Biosynthesis. Int J Mol Sci.

[39] Wang T, Yang Y, Lou S, Wei W, Zhao Z, Ren Y, Lin C, Ma L (2019). Genome-Wide Characterization and Gene Expression Analyses of GATA Transcription Factors in Moso Bamboo (*Phyllostachys edulis*). Int J Mol Sci.

[40] Zhu W, Guo Y, Chen Y, Wu D, Jiang L (2020). Genome-wide identification, phylogenetic and expression pattern analysis of GATA family genes in *Brassica napus*. BMC Plant Biol.

[41] Zhang K, Jia L, Yang D, Hu Y, Njogu MK, Wang P, Lu X, Yan C. Genome-Wide Identification, Phylogenetic and Expression Pattern Analysis of GATA Family Genes in Cucumber (*Cucumis sativus* L.). Plants*.* 2021; 10(8):1626. 10.3390/plants10081626 PMC8401448 34451671

[42] Strayer C, Oyama T, Schultz TF, Raman R, Somers DE, Mas P, Panda S, Kreps JA, Kay SA (2000). Cloning of the *Arabidopsis* clock gene *TOC1*, an autoregulatory response regulator homolog. Science.

[43] Shikata M, Matsuda Y, Ando K, Nishii A, Takemura M, Yokota A, Kohchi T (2004). Characterization of *Arabidopsis ZIM*, a member of a novel plant-specific GATA factor gene family. J Exp Bot.

[44] Casson S, Spencer M, Walker K, Lindsey K (2005). Laser capture microdissection for the analysis of gene expression during embryogenesis of Arabidopsis. Plant J.

[45] Richter R, Bastakis E, Schwechheimer C (2013). Cross-repressive interactions between SOC1 and the GATAs GNC and GNL/CGA1 in the control of greening, cold tolerance, and flowering time in Arabidopsis. Plant Physiol.

[46] Chiang YH, Zubo YO, Tapken W, Kim HJ, Lavanway AM, Howard L, Pilon M, Kieber JJ, Schaller GE (2012). Functional characterization of the GATA transcription factors GNC and CGA1 reveals their key role in chloroplast development, growth, and division in Arabidopsis. Plant Physiol.

[47] Mara CD, Irish VF (2008). Two GATA transcription factors are downstream effectors of floral homeotic gene action in Arabidopsis. Plant Physiol.

[48] Huang H, Gao X, Gao X, Zhang S, Zheng Y, Zhang N, Hong B, Zhao X, Gu Z (2022). Flower color mutation, pink to orange, through CmGATA4 - CCD4a-5 module regulates carotenoids degradation in chrysanthemum. Plant Sci.

[49] Shen C, Zhang Y, Li Q, Liu S, He F, An Y, Zhou Y, Liu C, Yin W, Xia X (2021). *PdGNC* confers drought tolerance by mediating stomatal closure resulting from NO and H_2_O_2_ production via the direct regulation of *PdHXK1* expression in *Populus*. New Phytol.

[50] Wang Y, Cao X, Zhang D, Li Y, Wang Q, Ma F, Xu X, Zhan X, Hu T (2023). SlGATA17, A tomato GATA protein, interacts with SlHY5 to modulate salinity tolerance and germination. Environ Exp Bot.

[51] Hu S, Hou Y, Zhao L, Zheng Y, Jin P (2023). Exogenous 24-epibrassinolide alleviates chilling injury in peach fruit through modulating PpGATA12-mediated sucrose and energy metabolisms. Food Chem.

[52] Mistry J, Chuguransky S, Williams L, Qureshi M, Salazar GA, Sonnhammer ELL, Tosatto SCE, Paladin L, Raj S, Richardson LJ, Finn RD, Bateman A (2021). Pfam: The protein families database in 2021. Nucleic Acids Res.

[53] Jaillon O, Aury JM, Noel B, Policriti A, Clepet C, Casagrande A, Choisne N, Aubourg S, Vitulo N, Jubin C, Vezzi A, Legeai F, Hugueney P, Dasilva C, Horner D, Mica E, Jublot D, Poulain J, Bruyère C, Billault A, Segurens B, Gouyvenoux M, Ugarte E, Cattonaro F, Anthouard V, Vico V, Del Fabbro C, Alaux M, Di Gaspero G, Dumas V, Felice N, Paillard S, Juman I, Moroldo M, Scalabrin S, Canaguier A, Le Clainche I, Malacrida G, Durand E, Pesole G, Laucou V, Chatelet P, Merdinoglu D, Delledonne M, Pezzotti M, Lecharny A, Scarpelli C, Artiguenave F, Pè ME, Valle G, Morgante M, Caboche M, Adam-Blondon AF, Weissenbach J, Quétier F, Wincker P (2007). The grapevine genome sequence suggests ancestral hexaploidization in major angiosperm phyla. Nature.

[54] Eddy SR (1998). Profile hidden Markov models. Bioinformatics.

[55] Letunic I, Khedkar S, Bork P (2021). SMART: recent updates, new developments and status in 2020. Nucleic Acids Res.

[56] Lu S, Wang J, Chitsaz F, Derbyshire MK, Geer RC, Gonzales NR, Gwadz M, Hurwitz DI, Marchler GH, Song JS, Thanki N, Yamashita RA, Yang M, Zhang D, Zheng C, Lanczycki CJ, Marchler-Bauer A (2020). CDD/SPARCLE: the conserved domain database in 2020. Nucleic Acids Res.

[57] Artimo P, Jonnalagedda M, Arnold K, Baratin D, Csardi G, de Castro E, Duvaud S, Flegel V, Fortier A, Gasteiger E, Grosdidier A, Hernandez C, Ioannidis V, Kuznetsov D, Liechti R, Moretti S, Mostaguir K, Redaschi N, Rossier G, Xenarios I, Stockinger H (2012). ExPASy: SIB bioinformatics resource portal. Nucleic Acids Res.

[58] Horton P, Park KJ, Obayashi T, Fujita N, Harada H, Adams-Collier CJ, Nakai K (2007). WoLF PSORT: protein localization predictor. Nucleic Acids Res.

[59] Crooks GE, Hon G, Chandonia JM, Brenner SE (2004). WebLogo: a sequence logo generator. Genome Res.

[60] Tamura K, Stecher G, Kumar S (2021). MEGA11: Molecular Evolutionary Genetics Analysis Version 11. Mol Biol Evol.

[61] Wang Y, Tang H, Debarry JD, Tan X, Li J, Wang X, Lee TH, Jin H, Marler B, Guo H, Kissinger JC, Paterson AH (2012). MCScanX: a toolkit for detection and evolutionary analysis of gene synteny and collinearity. Nucleic Acids Res.

[62] Krzywinski M, Schein J, Birol I, Connors J, Gascoyne R, Horsman D, Jones SJ, Marra MA (2009). Circos: an information aesthetic for comparative genomics. Genome Res.

[63] Chen C, Chen H, Zhang Y, Thomas HR, Frank MH, He Y, Xia R (2020). TBtools: An Integrative Toolkit Developed for Interactive Analyses of Big Biological Data. Mol Plant.

[64] Gaut BS, Morton BR, McCaig BC, Clegg MT (1996). Substitution rate comparisons between grasses and palms: synonymous rate differences at the nuclear gene Adh parallel rate differences at the plastid gene rbcL. Proc Natl Acad Sci U S A.

[65] Hu B, Jin J, Guo AY, Zhang H, Luo J, Gao G. GSDS 2.0: an upgraded gene feature visualization server. Bioinformatics*.* 2015; 31(8):1296–7. 10.1093/bioinformatics/btu817 PMC4393523 25504850

[66] Bailey TL, Boden M, Buske FA, Frith M, Grant CE, Clementi L, Ren J, Li WW, Noble WS (2009). MEME SUITE: tools for motif discovery and searching. Nucleic Acids Res.

[67] Lescot M, Déhais P, Thijs G, Marchal K, Moreau Y, Van de Peer Y, Rouzé P, Rombauts S (2002). PlantCARE, a database of plant *cis*-acting regulatory elements and a portal to tools for *in silico* analysis of promoter sequences. Nucleic Acids Res.

[68] Livak KJ, Schmittgen TD (2001). Analysis of Relative Gene Expression Data Using Real-Time Quantitative PCR and the 2^−ΔΔCT^ Method. Methods.

[69] Zhang X, Zhang L, Ji M, Wu Y, Zhang S, Zhu Y, Yao J, Li Z, Gao H, Wang X. Genome-wide identification and expression analysis of the B-box transcription factor gene family in grapevine (*Vitis vinifera* L.). BMC Genomics*.* 2021; 22(1):221. 10.1186/s12864-021-07479-4 PMC8008696 33781207

