# Supplementary figures and images for: Analysis of GATA transcription factors and their expression patterns under abiotic stress in grapevine (Vitis vinifera L.)
Source: BMC Plant Biol. 2023 Dec 2;23:611. doi: 10.1186/s12870-023-04604-1 (PMC10693065; doi:10.1186/s12870-023-04604-1)

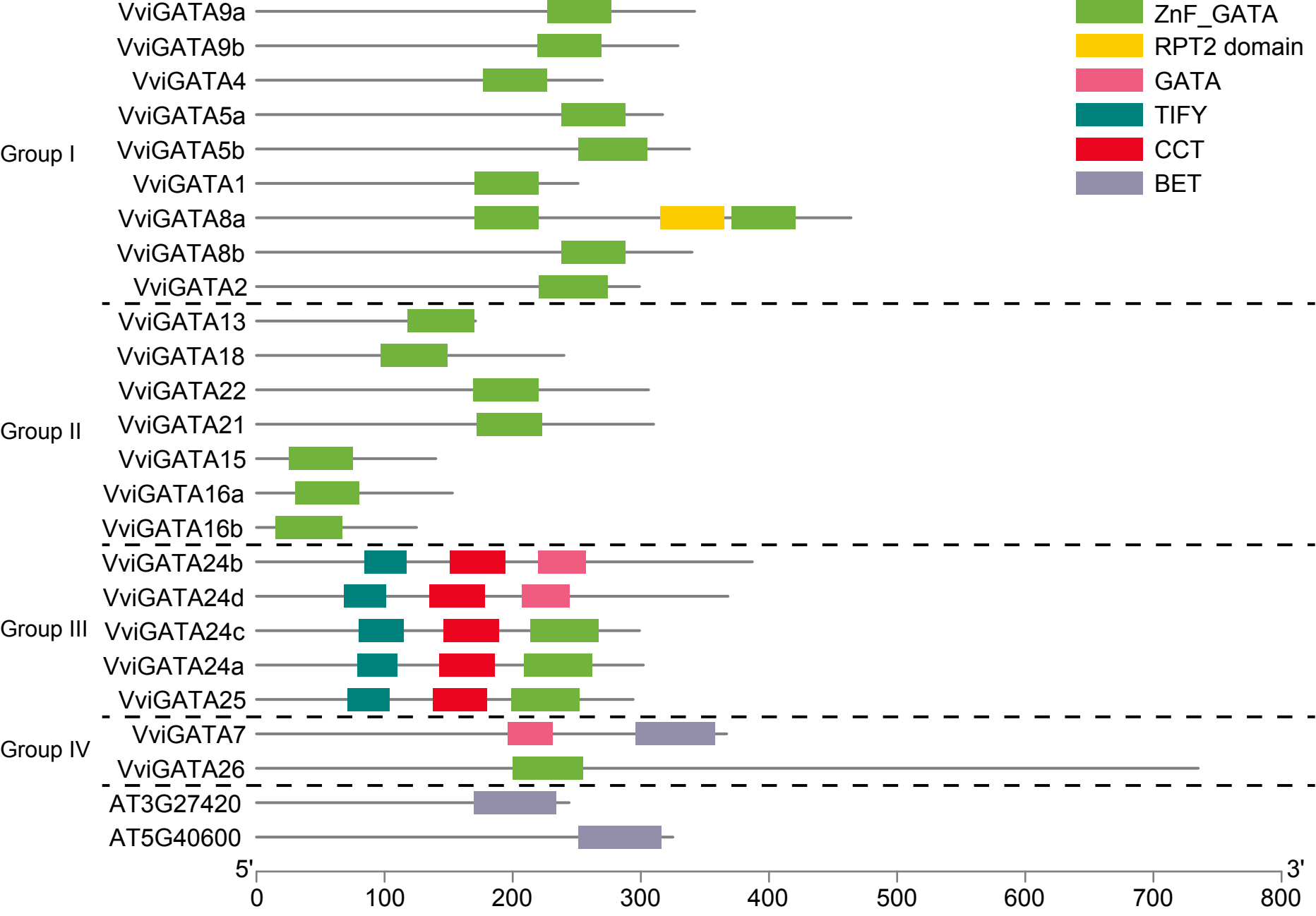

Supplement: Supplementary file 4 — Additional file 4: Fig. S2. Distribution of conserved GATA domains in VviGATA proteins. [file 12870_2023_4604_MOESM4_ESM.pdf]

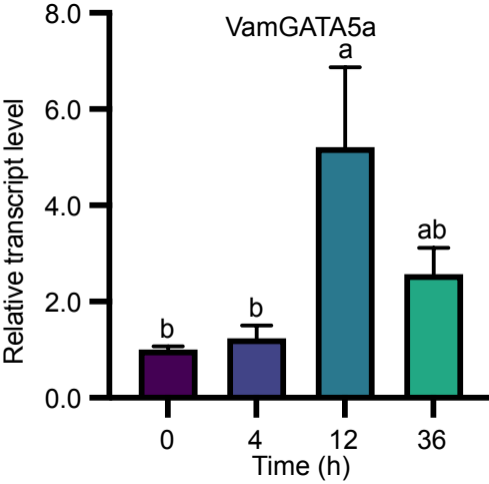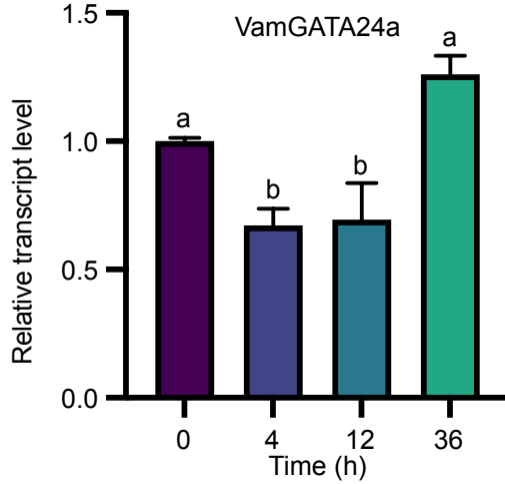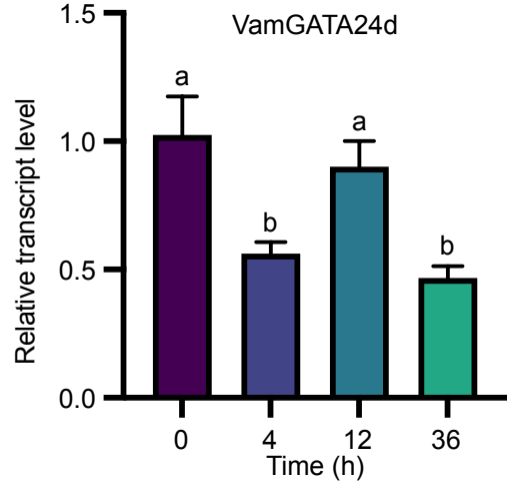

Supplement: Supplementary file 8 — Additional file 8: Fig. S2. Alignment of the coding sequences of five cloned GATA genes form Vitis amurensis ‘Shuangyou’. [file 12870_2023_4604_MOESM8_ESM.pdf]
