# Supplementary material for: Analysis of GATA transcription factors and their expression patterns under abiotic stress in grapevine (Vitis vinifera L.)
Source: BMC Plant Biol. 2023 Dec 2;23:611. doi: 10.1186/s12870-023-04604-1 (PMC10693065; doi:10.1186/s12870-023-04604-1)
Supplement: Supplementary file 5 — Additional file 5: Table S3. GATA protein motif sequences identified using the MEME tool. [file 12870_2023_4604_MOESM5_ESM.docx]

TableS3 GATA protein motif sequences identified using the MEME tool

| Name | Logo | Sequences | E-value | Sites | Width |
| --- | --- | --- | --- | --- | --- |
| Motif1 |  | TPLWRTGPAGPKSLCNACGIR | 1.3e-334 | 23 | 21 |
| Motif2 |  | YKSGRLVPEYRPAASPTFVSEKHSNSHRKVLEMRRQK | 9.0e-172 | 9 | 37 |
| Motif3 |  | RSNLPHRIASLIRFREKRKERCFDKKIRYTVRKEVALRMQRKKGQFTSSK | 2.8e-105 | 5 | 50 |
| Motif4 |  | RRCAHCGTTK | 8.0e-058 | 22 | 10 |
| Motif5 |  | TNQLTLSFZGEVYVFDAVTPEKVQAVLLLLGGREVP | 1.4e-055 | 5 | 36 |
| Motif6 |  | LCVPADDLAELEWLSNFVEDSFS | 2.0e-045 | 7 | 23 |
| Motif7 |  | KQRRKLGEEEEAAILLMALSCGLVY | 1.2e-028 | 5 | 25 |
| Motif8 |  | WANKGTLRDLSK | 4.6e-017 | 6 | 12 |
| Motif9 |  | FPAKARSKRPRASPRDW | 7.5e-015 | 8 | 17 |
| Motif10 |  | GDHFSIDDLLDFSND | 1.3e-014 | 6 | 15 |
| Motif11 |  | NGDDYLIHQHAGPDFRHLI | 2.4e-005 | 2 | 19 |
| Motif12 |  | KWMSSKMRLMRKMMNSDCT | 1.7e-004 | 2 | 19 |
| Motif13 |  | HQQQQQQZLYHHNFVF | 2.3e-003 | 3 | 16 |
