# Supplementary material for: Analysis of GATA transcription factors and their expression patterns under abiotic stress in grapevine (Vitis vinifera L.)
Source: BMC Plant Biol. 2023 Dec 2;23:611. doi: 10.1186/s12870-023-04604-1 (PMC10693065; doi:10.1186/s12870-023-04604-1)
Supplement: Supplementary file 3 — Additional file 3: Fig. S1. Alignment of conserved GATA domains from the 23 VviGATA transcription factors. [file 12870_2023_4604_MOESM3_ESM.pdf]

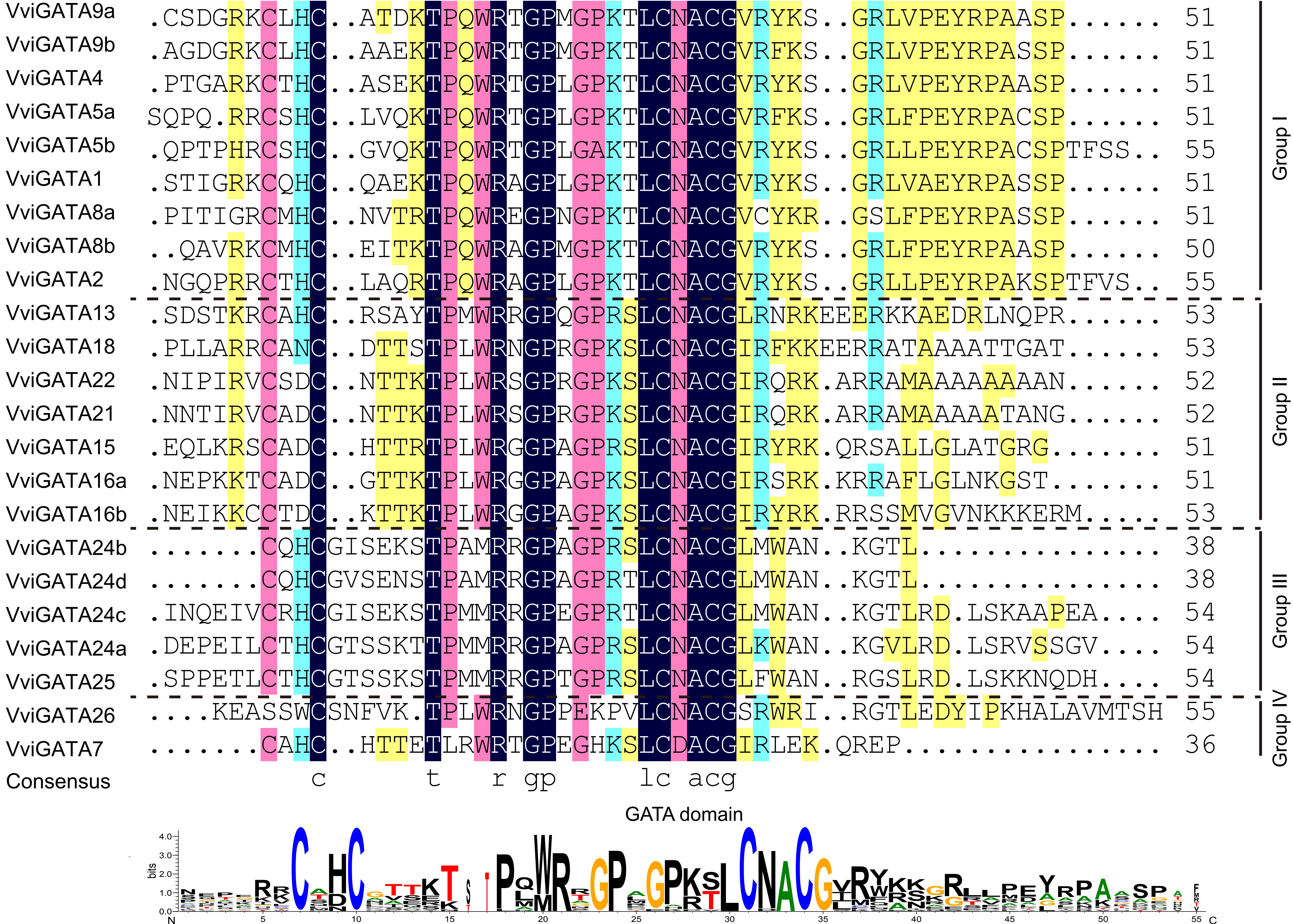
VviGATA9a .CSDGRKCLHC..ATDKTPQWRTGPMGPKTLCNACGVRYKS..GRLVPEYRPAASP...... 51
VviGATA9b .AGDGRKCLHC..AAEKTPQWRTGPMGPKTLCNACGVRFKS..GRLVPEYRPASSP...... 51
VviGATA4 .PTGARKCTHC..ASEKTPQWRTGPLGPKTLCNACGVRYKS..GRLVPEYRPAASP...... 51
VviGATA5a SQPQ.RRCSHC..LVQKTPQWRTGPLGPKTLCNACGVRFKS..GRLFPEYRPACSP...... 51
VviGATA5b .QPTPHRCSHC..GVQKTPQWRTGPLGAKTLCNACGVRYKS..GRLLPEYRPACSPTFSS.. 55
VviGATA1 .STIGRKCQHC..QAEKTPQWRAGPLGPKTLCNACGVRYKS..GRLVAEYRPASSP...... 51
VviGATA8a .PITIGRCMHC..NVTRTPQWREGPNGPKTLCNACGVCYKR..GSLFPEYRPASSP...... 51
VviGATA8b ..QAVRKCMHC..EITKTPQWRAGPMGPKTLCNACGVRYKS..GRLFPEYRPAASP...... 50
VviGATA2 .NGQPRRCTHC..LAQRTPQWRAGPLGPKTLCNACGVRYKS..GRLLPEYRPAKSPTFVS.. 55
VviGATA13 .SDSTKRCAHC..RSAYTPMWRRGPQGPRSLCNACGLRNRKEEERKKAEDRLNQPR...... 53
VviGATA18 .PLLARRCANC..DTTSTPLWRNGPRGPKSLCNACGIRFKKEERRATAAAATTGAT...... 53
VviGATA22 .NIPIRVCSDC..NTTKTPLWRSGPRGPKSLCNACGIRQRK.ARRAMAAAAAAAN...... 52
VviGATA21 .NNTIRVCADC..NTTKTPLWRSGPRGPKSLCNACGIRQRK.ARRAMAAAAATANG...... 52
VviGATA15 .EQLKRSCADC..HTTRTPLWRGGPAGPRSLCNACGIRYRK.QRSALLGLATGRG....... 51
VviGATA16a .NEPKKTCADC..GTTKTPLWRGGPAGPKSLCNACGIRSRK.KRRAFLGLNKGST....... 51
VviGATA16b .NEIKKCCTDC..KTTKTPLWRGGPAGPKSLCNACGIRYRK.RRSSMVGVNKKKERM..... 53
VviGATA24b ......CQHCGISEKSTPAMRRGPAGPRSLCNACGLMWAN..KGTL.............. 38
VviGATA24d ......CQHCGVSENSTPAMRRGPAGPRTLCNACGLMWAN..KGTL.............. 38
VviGATA24c .INQEIVCRHCGISEKSTPMMRRGPEGPRTLCNACGLMWAN..KGTLRD.LSKAAPEA.... 54
VviGATA24a .DEPEILCTHCGTSSKTTPMMRRGPAGPRSLCNACGLKWAN..KGVLRD.LSRVSSGV.... 54
VviGATA25 .SPPETLCTHCGTSSKSTPMMRRGPTGPRSLCNACGLFWAN..RGSLRD.LSKKNQDH.... 54
VviGATA26 ....KEASSWCSNFVK.TPLWRNGPPEKPVLCNACGSRWRI..RGTLEDYIPKHALAVMTSH 55
VviGATA7 ......CAHC..HTTETLRWRTGPEGHKSLCDACGIRLEK.QREP............... 36
Consensus c t r gp lc acg
Group I
Group II
Group III
Group IV
GATA domain
bits
N
C
