# Supplementary material for: Analysis of GATA transcription factors and their expression patterns under abiotic stress in grapevine (Vitis vinifera L.)
Source: BMC Plant Biol. 2023 Dec 2;23:611. doi: 10.1186/s12870-023-04604-1 (PMC10693065; doi:10.1186/s12870-023-04604-1)
Supplement: Supplementary file 9 — Additional file 9: Fig. S3. Real-Time Quantitative PCR analysis of expression of selected VviGATA genes. The grapevine ACTIN1 gene was used as an internal control to normalize expression levels. Letters indicate significance differences in gene expression using one-way ANOVA, followed by Fisher's least significant difference method (p < 0.05). [file 12870_2023_4604_MOESM9_ESM.pdf]

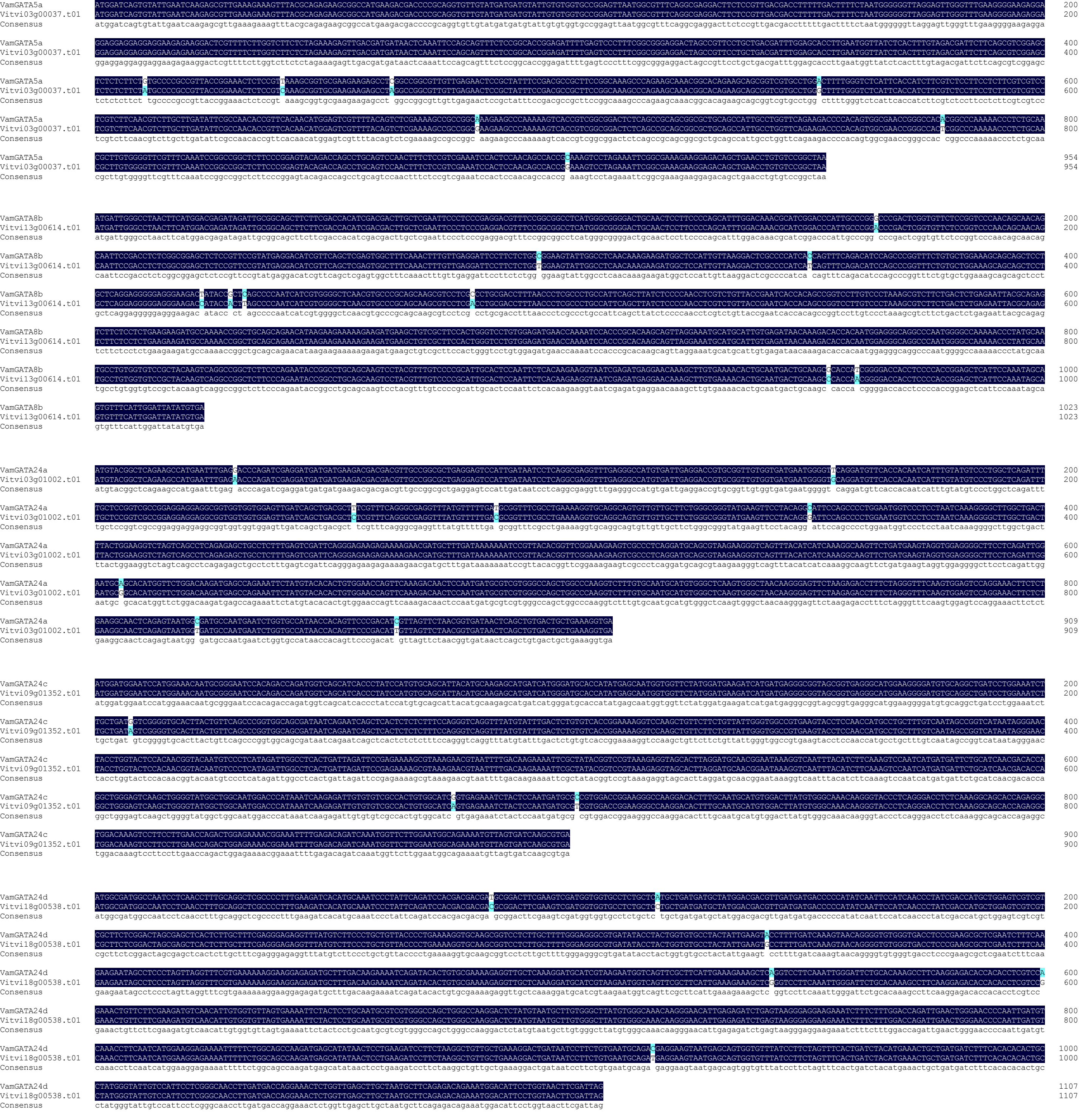
VamGATA5a
Vitvi03g00037.t01
Consensus
VamGATA8b
Vitvi13g00614.t01
VamGATA24a
Vitvi03g01002.t01
VamGATA24c
Vitvi09g01352.t01
VamGATA24d
Vitvi18g00538.t01
